# Microvascular free flaps in head and neck reconstruction: an analysis of outcomes in elderly patients

**DOI:** 10.1007/s00784-025-06262-z

**Published:** 2025-03-18

**Authors:** Jakob Fenske, Philipp Lampert, Claudius Steffen, Henri Kreiker, Steffen Koerdt, Kilian Kreutzer, Max Heiland, Carsten Rendenbach

**Affiliations:** Department of Oral and Maxillofacial Surgery, Charité Universitätsmedizin Berlin, Corporate Member of Freie Universität Berlin and Humboldt-Universität zu Berlin, Augustenburger Platz 1, 13353 Berlin, Germany

**Keywords:** Free flap surgery, Microvascular free flap, Elderly, Maxillofacial reconstruction, Complications, Delirium

## Abstract

**Objectives:**

Maxillofacial reconstruction using microvascular free flaps is a well-established procedure to restore facial defects across various patients. Elderly patients face increased perioperative risks due to higher prevalence of comorbidities. This study aims to evaluate the characteristics of patients aged ≥ 75 years and assess the impact of advanced age on outcomes in microvascular free flap reconstruction for head and neck defects.

**Methods:**

A retrospective analysis was conducted on patients who underwent microvascular free flap head and neck reconstruction between April 2017 and July 2023. After matching, patients aged ≥ 75 were compared to those < 75 years regarding comorbidities, surgical and treatment variables, complication rates and outcomes. Multivariate models were developed to test the influence of age on flap complications.

**Results:**

1050 patients met the inclusion criteria. 290 patients (28%) were ≥ 75 years old. Following case matching, 580 patients (276 females, mean age 74.1 ± 8.9 years) were included in the analysis. Patients ≥ 75 years had higher rates of comorbidities, co-medications and postoperative delirium. No significant differences were observed in flap complications between both groups in univariate analysis. Although one multivariate model suggested a potential non-linear effect of age on flap complications, this was not validated in another model.

**Conclusions:**

Despite the increased prevalence of comorbidities and perioperative risk factors, microvascular free flap reconstruction remains a safe and viable procedure for elderly patients when performed in a structured setting at a high-volume center, as complication rates do not appear to be directly influenced by age.

**Clinical relevance:**

Free Flap surgery is a safe procedure in elderly patients.

## Introduction

Microvascular free flaps have emerged as the gold standard for complex reconstruction cases in oral and maxillofacial surgery. These techniques offer a broad range of surgical options, ranging from soft tissue to osseous and compound flaps, providing versatility in addressing diverse clinical indications.

Similar to other surgical disciplines, patient demographics in head and neck reconstruction are diverse. While a significant proportion of patients, particularly in oncologic cases, are in their seventh decade of life [1], there is also a notable representation of even older patients [2]. This elderly cohort frequently presents with multiple comorbidities, ongoing medications, and other risk factors, which complicate perioperative management, particularly in relation to surgery and general anesthesia.

Previous studies have explored outcomes in elderly patients undergoing maxillofacial reconstruction, but many were limited by small sample sizes, lacked well-matched control groups [3] or mainly focused on oncological indications. Moreover, donor site complications remained underrepresented. Despite these limitations, the majority of studies have concluded that microvascular free flap surgery is generally safe in the elderly population [3–8]. However, some researchers have identified advanced age as a risk factor for increased postoperative complications and higher mortality rates [9, 10].

In this retrospective single-center cohort study, we characterize demographic and therapeutic profiles of elderly patients undergoing microvascular free flap surgery for maxillofacial reconstruction. Additionally, we employ a matched-pair analysis to analyze complication outcomes and patient characteristics, leveraging a large-scale database to provide robust, comparative insight into surgical outcomes in this population.

## Patients & methods

### Study cohort

Ethical approval was obtained by the local ethics committee (EA2/138/18). Patients who underwent reconstructive microvascular free flap surgery for head and neck defects between April 2017 and July 2023 at the Department of Oral and Maxillofacial Surgery at Charité Universitätsmedizin Berlin, Germany, were identified. The inclusion criteria were: (1) patients receiving microvascular free flaps (osseous and non-osseous) for (2) head and neck reconstruction, who were (3) at least 18 years of age at the time of surgery. Patients who received (1) a non-titanium osteosynthesis plate, (2) had missing documentation or (3) received more than one flap simultaneously were excluded. One patient, who committed suicide during the immediate postoperative hospital stay, was also excluded.

### Outcomes and analyzed variables

The primary endpoint was the occurrence of postoperative flap complications. Patient (sex, age, constitutional data, comorbidities), initial disease (tumor and peri-interventional specifications), and treatment characteristic (flap specifications, multimodal aspects, complications and treatment) as well as follow-up documentations were collected.

### Statistical analysis

All statistical calculations were performed using SPSS, Version 29 (IBM Corp.) and RStudio Version 2024.09.0 + 375. To account for potential confounders and improve comparability, we performed a matched-pair analysis. Each patient ≥ 75 years was matched with a patient < 75 years. Matching criteria included gender, indication (malignant vs. non-malignant), flap type (osseous vs. non-osseous) and follow-up time using a nearest neighbor matching algorithm without replacement. All eligible patients were successfully matched, and the final analysis was conducted using this matched cohort. Results were considered statistically significant if the p-value was < 0.05. Inclusion of the null value in the 95%-confidence interval (CI) of odds ratios (OR) was recorded as non-significant, while non-inclusion was recorded as significant. For qualitative variables, comparisons between patients ≥ 75 and < 75 years were made using the Chi-squared test. In cases where the expected cell counts were below five, Fisher’s exact test was used instead. For quantitative variables, the distribution was first assessed for normality using the Shapiro-Wilk test. Based on the results, the Mann-Whitney U test was conducted to compare medians between the groups. To assess the effect of age on flap complications continuously, a generalized additive model (GAM) and a segmented regression were employed using the “mgcv” [11] and “segmented” [12] packages in R. The GAM was fit using a binomial distribution with a logit link function to model the probability of flap complications, including age as a smoothed predictor to capture potential non-linear relationships. The segmented regression model was applied to identify a potential age threshold for increased flap complication risk with the initial breakpoint estimate set at 75 years and subsequently utilizing binomial logistic regression approach. Previously identified univariate predictors were included in both models to adjust for confounding.

## Results

### Patient characteristics

A total of 1050 patients met the inclusion criteria. 290 patients (28%) were at least 75 years old. After matching, 580 patients were included and divided in two groups: patients ≥ 75 years and < 75 years of age. The mean follow-up time was 21.5 ± 18.3 months, 276 (48%) patients were female. The mean age at surgery was 74.1 ± 8.9 years. Indications for surgery were malignant tumors (89%), benign tumors (0.3%), osteoradionecrosis (5%), medication associated osteonecrosis of the jaw (2%), osteomyelitis (1%) and others (3%). In univariate analysis, patients ≥ 75 years had a lower prevalence of preoperative history of smoking (18% vs. 29%; *p* < 0.001) and alcohol abuse (5% vs. 20%; *p* < 0.001), and a higher prevalence of diabetes mellitus (21% vs. 14%; *p* = 0.03), atherosclerosis (23% vs. 15%; *p* = 0.008), arterial hypertension (66% vs. 50%; *p* < 0.001), cardiac arrhythmia (20% vs. 7%; *p* < 0.001), and chronic kidney disease (8% vs. 3%; *p* = 0.004). The mean hospitalization was significantly longer in patients ≥ 75 years (18.7 vs. 17.3 days; *p* = 0.009). Antihypertensives (69% vs. 52%; *p* < 0.001) and anticoagulants (22% vs. 11%; *p* < 0.001) were administered more often in older patients (Table 1).

**Table 1:** Patient characteristics and comorbidities. (*OR = odds ratio*,* ci = confidence interval*,* bmi = body mass index*,* imc/icu = intermediate/intensive care unit*,* ckd = chronic kidney disease*)

Variable		≥ 75 Years No. (%)	< 75 Years No. (%)	*p*	OR [95%-CI]
Female		138 (48%)	138 (48%)	> 0.99	1.00 [0.72;1.39]
Age at surgery (years)		80.7 ± 4.4	67.7 ± 6.5	***< 0.001***	n.a.
BMI (kg/m^2^)		24.7 ± 4.2	25.4 ± 5.2	0.18	n.a.
Follow-up (months)		20.0 ± 18.0	23.0 ± 18.6	***0.04***	n.a.
Hospitalization (days)		18.7 ± 12.3	17.3 ± 12.4	***0.009***	n.a.
Days on IMC/ICU		2.8 ± 7.4	2.0 ± 3.4	0.64	n.a.
**Comorbidities**					
	Smoking	51 (18%)	85 (29%)	***< 0.001***	0.52 [0.35;0.76]
	Alcohol abuse	15 (5%)	58 (20%)	***< 0.001***	0.22 [0.12;0.40]
	Diabetes mellitus	61 (21%)	41 (14%)	***0.03***	1.62 [1.05;2.50]
	Arteriosclerosis	68 (23%)	43 (15%)	***0.008***	1.76 [1.15;2.69]
	Hyperlipidemia	33 (11%)	36 (12%)	0.70	0.91 [0.55;1.50]
	Arterial hypertension	192 (66%)	144 (50%)	***< 0.001***	1.99 [1.42;2.78]
	Cardiac arrhythmia	59 (20%)	21 (7%)	***< 0.001***	3.27 [1.93;5.55]
	CKD	24 (8%)	8 (3%)	***0.004***	3.18 [1.40;7.20]
	History of thrombosis	12 (4%)	6 (2%)	0.15	2.04 [0.76;5.52]
**Medication**					
	Statins	99 (34%)	80 (28%)	0.09	1.36 [0.96;1.94]
	Antihypertensives	199 (69%)	151 (52%)	***< 0.001***	2.01 [1.44;2.82]
	Anticoagulants	65 (22%)	31 (11%)	***< 0.001***	2.41 [1.52;3.84]
	Antiplatelet drugs	82 (28%)	67 (23%)	0.15	1.31 [0.90;1.91]

Surgery and treatment specific parameters.

Postoperative intravenous antibiotics were administered for one week, as well as prophylactic anticoagulation with low molecular weight heparins during hospitalization. Regularly, fibula free flaps, scapula free flaps, deep circumflex iliac artery free flaps, radial forearm free flaps, anterolateral thigh flaps, latissimus dorsi flaps and parascapular flaps were used. In 13 patients, less common flaps were used (soleus perforator flap, anteromedial thigh flap, thoracodorsal artery perforator flap, latissimus-serratus compound flap, medial and lateral arm flap). Radial forearm free flaps were less common in patients ≥ 75 years old (51% vs. 60%; *p* = 0.04), while latissimus dorsi free flaps (8% vs. 3%; *p* = 0.02) and anterolateral thigh flaps (17% vs. 10%; *p* = 0.01) were used significantly more often in this group (Table 2).

**Table 2:** Surgery and treatment specifications. (*SD = standard deviation*,* or = odds ratio*,* ci = confidence interval*,* N.a. = not applicable*)

	Variable	≥ 75 Years No. (%)	< 75 Years No. (%)	*p*	OR [95%-CI]
	Surgery duration (minutes)	468.8 ± 135.0	498.9 ± 148.8	0.06	n.a.
**Donor site**					
	Fibula free flap	48 (17%)	57 (20%)	0.33	0.81 [0.53;1.24]
	Scapula free flap	13 (4%)	7 (2%)	0.17	1.90 [0.75;4.83]
	Deep circumflex iliac artery free flap	6 (2%)	3 (1%)	0.51	2.02 [0.50;8.16]
	Radial forearm free flap	148 (51%)	173 (60%)	***0.04***	0.71 [0.51;0.98]
	Anterolateral thigh flap	49 (17%)	28 (10%)	***0.01***	1.90 [1.16;3.13]
	Latissimus dorsi free flap	23 (8%)	10 (3%)	***0.02***	2.41 [1.13;5.16]
	Parascapular free flap	0 (0%)	2 (1%)	0.50	n.a.
	Other	3 (1%)	10 (3%)	0.05	0.29 [0.08;1.08]
**Multimodal treatment**					
	Prior osseous free flap	17 (6%)	20 (7%)	0.61	0.84 [0.43;1.64]
	Prior soft tissue free flap	22 (8%)	23 (8%)	0.88	0.95 [0.52;1.75]
	Anamnestic radiotherapy	41 (14%)	50 (17%)	0.30	0.79 [0.50;1.24]
	Adjuvant radiotherapy	69 (24%)	77 (27%)	0.44	0.86 [0.59;1.26]
	Anamnestic chemotherapy	17 (6%)	32 (11%)	***0.03***	0.50 [0.27;0.93]
	Adjuvant chemotherapy	14 (5%)	22 (8%)	0.17	0.62 [0.31;1.23]
	Immunotherapy	12 (4%)	11 (4%)	0.83	1.10 [0.48;2.52]

Complication rates.

Occurrence of complications was divided in subgroups: general postsurgical complications and flap complications. Elderly patients showed higher rates of postoperative delirium (12% vs. 6%; *p* = 0.008). Other complication rates did not significantly differ between both groups (Table 3).

**Table 3:** Complication rates. (*OR = odds ratio*,* ci = confidence interval*,* pae = pulmonary artery embolism*)

Variable		≥ 75 Years No. (%)	< 75 Years No. (%)	*p*	OR [95%-CI]
**General postsurgical complications**					
	30-days postoperative exitus letalis	6 (2%)	7 (2%)	0.78	0.85 [0.28;2.57]
	Delirium	34 (12%)	16 (6%)	***0.008***	2.27 [1.23;4.22]
	Stroke	1 (0%)	1 (0%)	> 0.99	1.00 [0.06;16.06]
	Myocardial infarction	6 (2%)	4 (1%)	0.52	1.51 [0.42;5.41]
	PAE	4 (1%)	8 (3%)	0.24	0.49 [0.15;1.66]
	Pneumonia	19 (7%)	18 (6%)	0.87	1.06 [0.54;2.06]
**Flap complications**					
	Postoperative bleeding	25 (9%)	26 (9%)	0.88	0.96 [0.54;1.70]
	Anastomosis revision	16 (6%)	13 (4%)	0.57	0.80 [0.38;1.70]
	Flap loss	10 (3%)	16 (6%)	0.23	0.61 [0.27;1.37]
	Wound healing disorder	74 (26%)	69 (24%)	0.63	1.10 [0.75;1.60]
	Infection	30 (10%)	23 (8%)	0.31	1.34 [0.76;2.37]
	Partial necrosis	23 (8%)	18 (6%)	0.42	1.30 [0.69;2.47]

### Multivariate models

In the multivariate models, the influence of age on the occurrence of any and specific flap complications was assessed including previously identified univariate predictors and used flap type (osseous vs. non-osseous). The GAM analysis indicated a non-linear effect of age on the probability of any flap complication. The segmented regression model estimated a breakpoint at 71.9 years, indicating a change in the relationship between age and risk of any flap complication at this age. The continuous age predictor did not show a significant increase in complications with age. The segmented model confirmed the influence of hospital stay (*p* = 0.004), alcohol abuse (*p* = 0.01), and follow-up duration (*p* = 0.003) as relevant predictors. As for the other specific flap complications, age was not identified as a relevant predictor by both models. Hospital stay, follow-up duration, osseous flaps and alcohol abuse were identified as more robust predictors for specific complications unanimously by both models. Table 4 presents the results of both models for all complications.

**Table 4:** Results of the generalized additive models and segmented regressions. (*WHD = wound healing disorder*,* ckd = chronic kidney disease*)

Variable	Any Complication		Postoperative Bleeding		Anastomosis Revision		Flap Loss		WHD		Infection		Partial Necrosis	
	Estimate	*p*	Estimate	*p*	Estimate	*p*	Estimate	*p*	Estimate	*p*	Estimate	*p*	Estimate	*p*
**Generalized Additive Models**														
Age (smoothed term)	-	***0.02***	-	0.34	-	0.99	-	0.49	-	0.07	-	0.50	-	0.26
Osseous Flap	-0.03	0.91	-0.29	0.50	-0.08	0.88	1.26	***0.008***	-0.06	0.83	1.42	***0.001***	-0.23	0.60
Hospitalization (days)	0.02	***0.004***	0.03	***0.004***	0.04	***0.002***	0.03	***0.005***	0.02	***0.003***	0.01	0.26	0.02	0.14
Smoking	-0.05	0.83	-0.03	0.94	0.01	0.99	-0.62	0.29	-0.24	0.40	-0.33	0.45	-0.36	0.45
Alcohol abuse	0.83	***0.009***	0.16	0.77	-1.26	0.26	1.50	***0.02***	0.78	***0.02***	0.65	0.24	1.32	***0.01***
Arterial hypertension	-0.25	0.35	-0.04	0.92	0.06	0.92	-0.45	0.49	-0.44	0.12	-0.15	0.75	0.05	0.91
Cardiac arrhythmia	-0.30	0.39	0.80	0.10	0.81	0.22	-0.99	0.25	0.05	0.88	-1.38	0.06	-0.47	0.52
Diabetes mellitus	0.12	0.63	0.39	0.30	-0.36	0.54	0.05	0.93	-0.03	0.90	-0.37	0.42	0.18	0.68
Arteriosclerosis	0.31	0.19	-0.07	0.87	0.27	0.60	0.82	0.09	0.33	0.20	0.76	***0.04***	0.23	0.60
CKD	0.18	0.66	-0.61	0.37	-0.58	0.50	-0.08	0.93	-0.52	0.29	0,55	0.36	-0.13	0.88
Anticoagulants	-0.11	0.72	0.27	0.58	-0.06	0.94	0.53	0.41	-0.30	0.39	0.39	0.43	-0.29	0.63
Antihypertensives	0.08	0.78	0.02	0.97	-0.04	0.96	0.17	0.80	0.32	0.28	0.06	0.89	-0.58	0.23
Follow-up (months)	0.02	***0.003***	-0.01	0.47	-0.01	0.46	-0.01	0.58	0.01	***0.04***	0.01	0.06	0.02	***0.04***
**Segmented Regressions**														
Estimated breakpoint (years)	71.9	-	79.0	-	62.9	-	69.0	-	69.0	-	62.0	-	75.0	-
Age	0.05	0.1	-0.03	0.16	21.48	0.84	0.14	0.42	0.10	0.14	0.17	0.67	0.04	0.35
Age (post-breakpoint effect)	-0.04	-	0.10	-	-2150	-	-0.23	-	-0.10	-	-0.16	-	-0.04	-
Osseous Flap	-0.07	0.77	-0.03	0.55	-0.12	0.80	1.17	***0.02***	-0.12	0.63	1.41	***0.001***	-0.28	0.53
Hospitalization (days)	0.02	***0.004***	0.03	***0.003***	0.04	***0.002***	0.03	***0.004***	0.02	***0.004***	0.01	0.28	0.02	0.16
Smoking	-0.06	0.81	-0.08	0.87	0.01	0.99	-0.61	0.30	-0.24	0.39	-0.31	0.49	-0.37	0.44
Alcohol abuse	0.82	***0.01***	0.18	0.74	-1.35	0.23	1.48	***0.02***	0.78	***0.02***	0.60	0.28	1.31	***0.01***
Arterial hypertension	-0.26	0.33	-0.04	0.93	0.08	0.90	-0.49	0.46	-0.47	0.10	-0.15	0.75	0.03	0.95
Cardiac arrhythmia	-0.31	0.38	0.80	0.11	0.82	0.21	-1.04	0.23	0.04	0.91	-1.38	0.06	-0.49	0.50
Diabetes mellitus	0.12	0.62	0.39	0.30	-0.37	0.53	0.03	0.96	-0.02	0.93	-0.36	0.43	0.19	0.67
Arteriosclerosis	0.31	0.19	-0.05	0.90	0.28	0.58	0.83	0.10	0.32	0.21	0.77	***0.04***	0.22	0.61
CKD	0.21	0.62	-0.62	0.37	-0.55	0.58	-0.04	0.97	-0.49	0.31	0.56	0.35	-0.11	0.89
Anticoagulants	-0.11	0.75	0.27	0.57	-0.03	0.96	0.55	0.40	-0.29	0.40	0.41	0.41	-0.29	063
Antihypertensives	0.08	0.75	-0.01	0.99	-0.02	0.97	0.26	0.70	0.35	0.24	0.06	0.90	-0.57	0.24
Follow-up (months)	0.02	***0.003***	-0.01	0.51	-0.01	0.38	-0.01	0.55	0.01	***0.04***	0.01	0.07	0.02	***0.04***

## Discussion

Microvascular free flap reconstruction is the gold standard in complex head and neck reconstructions across a diverse patient age spectrum. This study provides insights into the outcomes of free flap surgery in elderly patients in a high-volume setting using one of the largest single-center databases to date.

While previous research has addressed microvascular reconstruction in elderly patients, common biases in most studies were highlighted as well [3]. Generally, most existing research relies on relatively small cohort studies, limiting generalizability of findings. Although this study’s retrospective design introduces similar potential biases, our investigation benefits from a comparatively large patient cohort. Additionally, all patients were treated in the course of six years, which is remarkable as well due to constantly evolving therapeutical guidelines in both maxillofacial surgery and neighboring disciplines. Hence, this study enables conclusions on free flap surgery in elderly patients based on contemporary concepts of broader medical practices. The age cut-off at 75 years aligns with most authors [5, 7, 10], facilitating comparability.

Regarding overall duration of hospitalization, elderly patients tend to stay over one day longer than younger counterparts. This previously described circumstance [13] may be attributed to the frequent need for prolonged care in older patients as well as the longer recovery time in multimorbid patients. Moreover, our data are focused on the hospitalization and do not account for many elderly patients needing further geriatric rehabilitation, preventing them from directly returning home. Nevertheless, given the marginal difference of just over one day, this finding holds limited practical significance for health economic considerations.

Another analyzed factor was the prevalence of comorbidities and co-medications in older patients. As expected, elderly patients exhibit significantly more comorbidities and co-medications, particularly focusing on cardiovascular, diabetic and renal conditions. Theoretically, this circumstance predisposes this cohort to wound healing disorders and/or inflammatory complications [14, 15]. Moreover, especially cardiovascular diseases increase therapeutical risks, also concerning the inevitable general anesthesia [16, 17]. Nevertheless, the overall time spent on intensive or intermediate care units did not significantly differ between both groups, likely due to rigorous preoperative risk assessment and careful anesthetic management.

Beyond that, the vast majority of general perioperative complications was largely comparable between both groups. However, rates of postoperative delirium were twice as high in older patients. Many authors assessed the risk of postoperative delirium in head and neck reconstructions with microvascular free flaps. Besides risk factors such as alcohol abuse, prolonged surgery duration, transplant revisions or poor nutritional status, advanced age was identified to increase the risk of delirium [18–21]. Our results support this association, as two thirds of delirium cases occurred in patients ≥ 75 years. Since this complication adversely affects the patients’ well-being and often prolongs hospitalization and intensive care dependency, this topic warrants further research regarding well-defined risk assessment and prevention strategies. Thereby, microvascular transplantations could advance to an even more low-risk procedure in elderly patients.

While many of the aforementioned attributes varied between older and younger patients, a different outcome regarding flap complications was observed. With the high prevalence of comorbidities and co-medications serving as substantial risk factors in the elderly cohort, a higher risk of flap complications such as wound healing disorders and infections following diabetic and vascular conditions or bleeding events following various co-medications could be expected. Nevertheless, the final surgical outcome remained constant throughout all categories. To assess the influence of age on all flap complications in a multivariate approach, two models were applied. While the GAM identified age to show a non-linear relationship with the occurrence of flap complications, the segmented regression did not confirm a statistically significant influence of age on flap complications above the calculated breakpoint. Conversely, both models identified alcohol abuse as a more robust predictor for the detection of flap complications, as well as duration of hospitalization and follow-up duration as significantly correlated to complications as well. An in-depth analysis of the specific complication types using both models underscored the subordinate role of age in predicting complications as both models unanimously confirmed age not to be a robust predictor. Additionally, this subgroup analysis revealed the known risk factor alcohol to be more relevant throughout both models regarding overall complications, flap loss, wound healing disorders and necrosis. Moreover, usage of osseous or non-osseous flaps did not correlate with overall complication risk in both models, but seemed to be relevant for flap loss and infection. Nevertheless, the analyzed cohort and the employed models were optimized for age analysis. Thus, conclusions on other predictors should be drawn from specified models. In summary, the data derived from the models indicate that age may play a partial role in the risk for overall flap complications but is not as model-independently robust in risk prediction as other factors. Hence, microvascular free flap surgery can mostly be considered a safe procedure regarding the overall outcome when performed in a structured setting with individual risk adaptation. These results confirm the observed tendencies of the vast majority of similar studies [3–8].

Future research should address the following major topics regarding free flap surgery in older patients: On the one hand, prevention strategies for postoperative delirium, e.g. identification of specific predictors or implementation of strategies for circadian rhythm normalization such as light therapy [22] not only on intensive care units, but also on normal wards should be evaluated. On the other hand, while objective outcomes remain untouched by age, our results do not cover quality of life aspects. Evaluating the influence of age in complex microvascular free flap surgery not only regarding surgical parameters, but also towards individual aspects concerning altered quality of life, involving potentially elevated perioperative stress levels, may provide physicians and patients with evidence for optimized decision making. Additionally, further maxillofacial specific research on preoperative rehabilitative measures, such as prehabilitation concepts known from other medical disciplines [23], could further accelerate the patients’ regeneration and minimize the need for postsurgical interventions to minimize hospital stay and improve well-being.

### Limitations

This study has several limitations. First, the follow-up period in the elderly cohort is minimally shorter compared to the younger group. Second, due to its retrospective design, inferring causation from the observed correlations is prevented. To mitigate potential biases, a matched-pair analysis and multivariate models were employed. However, due to arbitrary determination of matching criteria and analyzed variables, inherent potential confounding and selection biases cannot be ruled out completely. Third, all included patients were treated at a single center, consequently reducing generalizability of the results as therapeutical approaches between different departments may vary.

## Conclusions

Although elderly patients exhibit more presurgical risk factors, overall outcomes of microvascular free flap surgeries in the head and neck area can be regarded as a safe procedure when performed under thorough risk assessment in a structured setting in a high-volume center. The two multivariate models employed in this study did not unanimously confirm age to be a predictor of flap complications, although partial influences cannot be ruled out. Identification of prevention strategies for postoperative delirium and quality of life analyses remain important topics for future research.

## Data Availability

Data is provided within the manuscript. Raw data cannot be shared to protect study participant privacy as direct patient data were involved in the analysis.
